# Evaluating the potential impact of enhancing HIV treatment and tuberculosis control programmes on the burden of tuberculosis

**DOI:** 10.1098/rsif.2015.0146

**Published:** 2015-05-06

**Authors:** Leonid Chindelevitch, Nicolas A. Menzies, Carel Pretorius, John Stover, Joshua A. Salomon, Ted Cohen

**Affiliations:** 1 Department of Epidemiology of Microbial Diseases, Yale School of Public Health, New Haven, CT, USA; 2 Center for Health Decision Science, Harvard T. H. Chan School of Public Health, Boston, MA, USA; 3 Department of Global Health and Population, Harvard T. H. Chan School of Public Health, Boston, MA, USA; 4 Futures Institute, Glastonbury, CT, USA

**Keywords:** antiretroviral therapy, HIV/AIDS, mathematical model, sub-Saharan Africa, tuberculosis

## Abstract

HIV has fuelled increasing tuberculosis (TB) incidence in sub-Saharan Africa. Better control of TB in this region may be achieved directly through TB programme improvements and indirectly through expanded use of antiretroviral therapy (ART) among those with HIV. We used a mathematical model of TB and HIV in South Africa to examine the potential epidemiological impact in scenarios involving improvements in three dimensions of TB programmes: coverage, diagnosis and treatment effectiveness, as well as expanded ART use through broadened eligibility. We projected the effect of alternative scenarios on TB prevalence, incidence and TB-related mortality over 20 years. Of the three dimensions of TB programme improvement, expanding coverage would produce the greatest reduction in TB burden. Compared with current performance, combined TB programme improvements were projected to decrease TB incidence by 30% over 5 years and 46% over 20 years, and decrease TB-related mortality by 45% over 5 years and 69% over 20 years. Expanded ART eligibility was projected to decrease TB incidence by 22% over 5 years and 45% over 20 years, and TB-related mortality by 22% over 5 years and 50% over 20 years. We found that over a 20-year horizon, TB-specific and HIV-specific programme changes contribute equally to incidence reductions, whereas the TB-specific changes produce a majority of the mortality benefits. An aggressive expansion of ART alongside traditional TB-specific control measures has the potential to greatly reduce TB burden, with the different elements of a combined approach having a synergistic effect in reducing long-term TB incidence and mortality.

## 1. Introduction

HIV has led to a large increase in the incidence of tuberculosis (TB) in sub-Saharan Africa. There is consensus that additional TB control efforts, extending beyond those needed in other settings, must be adopted to control TB in areas of high HIV prevalence [1]. Among other options, expanded TB control activities may include more intensive case finding, improved diagnostics and more effective TB treatment.

While improvements to the TB control programme will reduce disease burden, other health interventions may also contribute to better TB outcomes. In particular, antiretroviral therapy (ART) to treat HIV has been shown to reduce TB risk, and a number of modelled analyses have suggested the potential for substantial reductions in TB burden associated with ART in settings with HIV-fuelled TB epidemics [2–7]. Recent research findings suggest additional benefits of ART, including the HPTN 052 trial results that demonstrated almost complete interruption of HIV transmission for patients on ART [8], and a meta-analysis revealing how the effect of ART on TB risk is modified by CD4 cell count [9]. In addition, the interventional options for TB and HIV control are changing, with the availability of improved TB diagnostics [10,11] and the promise of new TB drug regimens [12], as well as a growing commitment to expanded ART access [13,14]. A less welcome development is rising TB drug resistance in settings with high TB and HIV prevalence [15,16]. Given these changes in the scientific understanding, epidemiological features and programmatic context of HIV and TB control, it is useful to revisit the comparative effect of ART and traditional TB control interventions on TB outcomes in settings of high HIV burden. Expanded access and increased eligibility for ART have recently been predicted to be cost-effective for the control of HIV in the general population (or the high-risk population in concentrated epidemics) [17], and to produce a significant reduction of the TB burden in HIV-positive patients in a high-burden setting [18].

In this study, we use a mathematical model of TB and HIV to investigate how expanded ART provision, accounting for its impact on reducing HIV transmission, compares with more direct efforts to control TB through improved access to TB care, use of more sensitive diagnostics such as Xpert, and more effective TB treatment that could result from the introduction of new treatment regimens.

We developed a dynamic compartmental model of TB–HIV epidemiology, adapted from a previously published model [19]. The model, described in detail in the Material and methods, simulates the progression and transmission of both TB and HIV, as well as the effect of disease control activities on epidemiology and health outcomes. We used a Bayesian approach [20–22] to calibrate the model to available epidemiological data, combining data on historical trends in TB and HIV epidemiology with prior information about model parameters, updated based on recent research findings which include evidence on the impact of ART initiation on HIV transmission risks [8], the magnitude of reductions in TB incidence among HIV-infected individuals initiating ART [9] and the performance of new TB diagnostic technology [23]. We used the calibrated model to investigate the impact of changes in HIV and TB control policy, both separately and in combination, their differential contribution to various TB outcomes, and the time horizon over which benefits accrue, contrasting between short-term (5-year) and long-term (20-year) outcomes. We expect the results to be relevant to settings with a high burden of TB and HIV and rapid ART expansion.

## 2. Results

### 2.1. Modelled scenarios and outcomes

The annual TB incidence in South Africa rose from approximately 300 per 100 000 in 1990 to approximately 1000 per 100 000 in 2010 [24], an increase that has been attributed largely to the HIV epidemic. We modelled a baseline scenario, and two sets of alternative scenarios reflecting intensified strategies for TB and HIV control. The baseline scenario assumed continuation of current TB and HIV control policy and programme functioning. In this scenario, ART coverage under the 2010 eligibility criteria (all individuals with a CD4 count less than 350 cells per µl or active TB) plateaus at 80% by 2017. In addition, only smear microscopy is used for the diagnosis of TB and the national TB programme continues to perform at its current levels both in terms of patient coverage and treatment effectiveness.

The first set of alternative scenarios examined TB programme improvements along three dimensions: coverage, diagnosis and treatment. The second set of alternative scenarios examined ART expansion in terms of both eligibility and treatment scale-up. These alternative scenarios were designed so as to provide an upper bound on what might be possible through improvements in a given intervention area.

We compared the epidemiological outcomes of baseline and alternative intervention scenarios over short-term (5-year) and long-term (20-year) time horizons, and quantified the effects on TB prevalence, TB incidence, prevalence of multidrug-resistant TB (MDR-TB), and mortality in individuals with active TB.

In our baseline scenario, both the TB prevalence and TB incidence continue rising for 5 years before stabilizing around 1%. The annual TB mortality rate, which we defined to include mortality from HIV-related TB, essentially stays constant at around 400 deaths per 100 000, whereas the prevalence of MDR-TB continues to rise slowly over the entire time horizon, such that close to 5% of all active TB cases have MDR-TB after 20 years.

**Figure 1.**
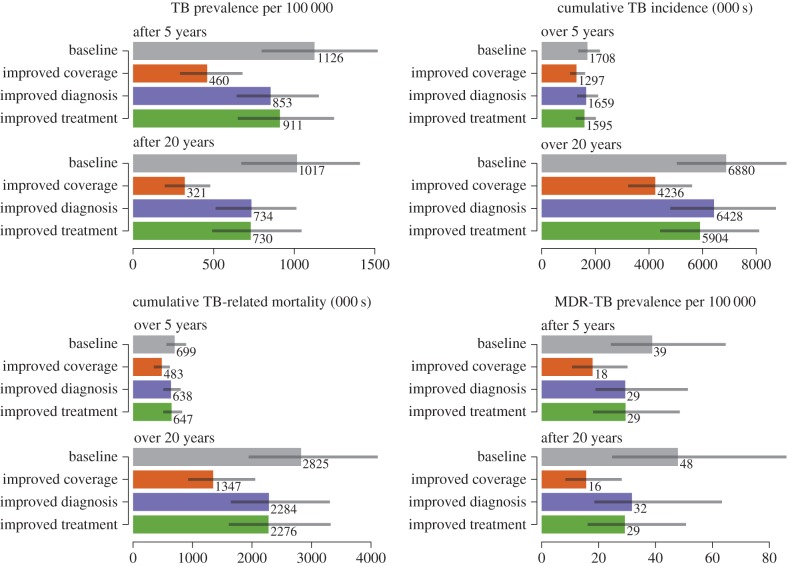
TB outcomes under different TB programme improvement strategies at 5 and 20 years. (Online version in colour.)

To understand the potential impact of improvements along three distinct dimensions of the TB programme (i.e. access, diagnosis and treatment), we first examined the separate effects of improving performance within each dimension (figure 1). Across the various outcome measures, the relative benefits owing to improvements in access to care were two to eight times greater than those owing to improved diagnostics, and two to four times greater than those owing to improvements in treatment.

**Figure 2.**
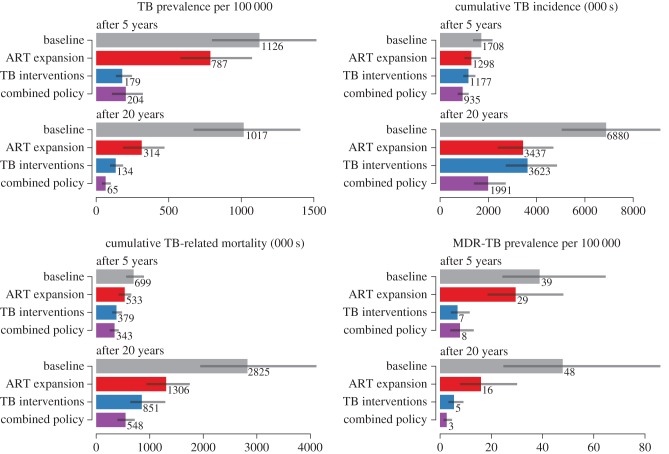
TB outcomes under different scenarios at 5 and 20 years. (Online version in colour.)

We also considered ART programme expansion, which combines universal ART eligibility with an increase in the rate of ART initiation (by 0.5 per year) for those eligible for treatment. We found that the potential benefits of ART scale-up were comparable to the combined effects of the direct TB programme improvements in the first set of alternative scenarios (figure 2). Expanded ART was predicted to reduce TB incidence and mortality by almost a quarter relative to the baseline over 5 years, and by nearly half over 20 years. The reduction in TB incidence and mortality produced by combining all TB programme improvements exceeds the effects of ART expansion for all outcomes over 20 years except for incidence, in which ART expansion has a larger effect.

**Figure 3.**
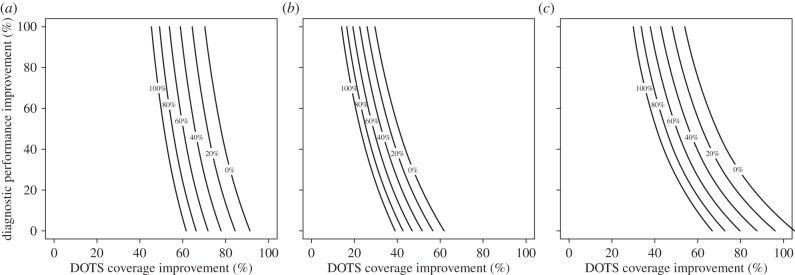
Isocline plots of the TB interventions needed to match ART expansion on (*a*) cumulative 5-year incidence, (*b*) cumulative 20-year incidence and (*c*) cumulative 5-year mortality. The axes represent the percentage improvement in DOTS coverage and diagnostic performance and the lines are labelled with the percentage improvement in clinical performance needed to achieve the same outcome as ART expansion. On every dimension, 0% corresponds to the baseline level and 100% to the maximum attainable level of improvement.

To consider the potential for combinations of programme improvements in both TB and HIV control, we conducted a threshold analysis in which we assessed the levels of improvement in direct TB programme outcomes required to produce equivalent overall benefits to those expected from scale-up of ART. We created three isocline plots (figure 3) indicating the degree of TB expansion along different dimensions required to match the ART expansion impact on the following outcomes: (i) short-term TB incidence, (ii) long-term TB incidence, and (iii) short-term TB-related mortality. Results are not shown for long-term TB incidence, as we found that for this outcome the impact produced by expanded ART programmes could not be matched even at the maximum level of improvement along each of the three dimensions of TB expansion.

**Figure 4.**
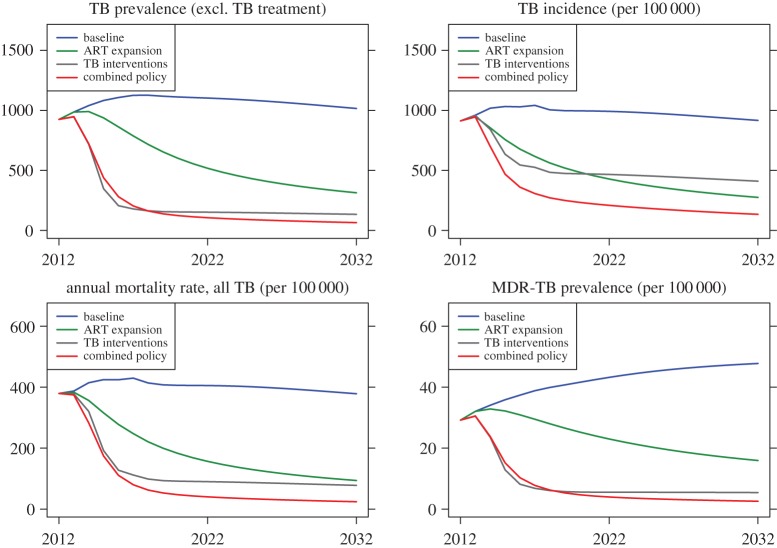
Selected metrics, comparison between the baseline, TB expansion, ART expansion and ART + TB scenarios. (Online version in colour.)

Figure 4 shows the projected outcomes of the baseline, ART programme expansion, maximally implemented TB programme improvement and a combined ART/TB programme expansion scenario. Our results suggest that an aggressive approach to TB programme expansion results in large early reductions in TB prevalence and TB-related mortality. These dramatic reductions in TB prevalence lead to subsequent declines in TB incidence as a result of decreased transmission. While control of TB continues to advance over the 20-year horizon under assumptions of a greatly improved TB control programme, the vast majority of the benefit occurs within the first decade of the change in programme performance, after which the level of benefit appears to plateau.

In contrast, ART programme expansion results in improvement in TB outcomes that accrues progressively over the 20-year horizon. While TB programme expansion results in a greater reduction of TB prevalence, ART expansion results in a greater reduction of TB incidence (figure 4). The impacts of the interventions also differ according to the patients’ HIV status. Thus, the incidence of TB in HIV-negative patients is reduced more effectively with the TB programme expansion than with ART expansion, whereas the incidence of TB in HIV-positive patients is reduced more effectively with ART expansion than with the TB programme expansion, at both 5 and 20 years. Both TB mortality and MDR-TB prevalence are projected to attain lower values under TB programme expansion, but the trend suggests that the long-term equilibrium MDR-TB prevalence may be similar for both interventions. The fraction of prevalent TB cases that are MDR remained in the 95% credible interval between 2% and 5% after 5 years, and between 3% and 7% after 20 years, regardless of the particular scenario we investigated. Finally, combining ART and TB programme expansion strategies leads to a dramatic decline of the TB epidemic over the 20-year horizon.

### 2.2. Sensitivity analysis

To quantify the uncertainty in our predictions, we constructed 95% credible intervals around each of our model outcomes. These credible intervals are shown in figures 1 and 2, as well as in electronic supplementary material, table S2. The corresponding 95% credible intervals on each of the model parameters are shown in electronic supplementary material, table S3. Our qualitative conclusions are robust to parameter uncertainty, as the rank order of the scenarios we considered with respect to each outcome remained unchanged for the majority of the resampled parameter sets. For instance, over 99% of all our parameter sets support the conclusion that TB programme improvements are more effective than ART expansion in reducing long-term TB prevalence and mortality, but less effective in reducing long-term TB incidence.

In order to further evaluate the robustness of our results, we performed several sensitivity analyses. We computed partial rank correlation coefficients of the decline in long-term TB incidence relative to the status quo with each of the model parameters, and examined the 10 most influential model parameters for this decline in TB care accessibility, diagnostic tool improvement, TB programme improvement and ART expansion (electronic supplementary material, figure S2). We also examined the effect of the time required to bring TB interventions to scale. We found that if TB programme scale-up requires 5 years instead of 2, the absolute reductions in long-term TB incidence and mortality with respect to the baseline each drop by 6%. If TB programme scale-up requires 10 years instead of 2, the reductions each drop by 14%. We also examined the sensitivity of results to more conservative assumptions about ART effectiveness for preventing HIV transmission, and about the rate of ART scale-up under the ART expansion scenario. If the effectiveness of ART for preventing HIV transmission were halved from 96% to 48%, the reductions in long-term TB incidence and mortality with respect to the baseline would decrease by 9% and 7%, respectively. If the rate of ART scale-up under the ART expansion scenario were halved, the reductions in TB incidence and mortality projected for this scenario would each drop by 15%.

## 3. Discussion

In this work, we examined the potential impact of different control measures on the TB epidemic in a high-burden setting. These measures included direct improvements in the TB control programme in terms of coverage, diagnosis and treatment effectiveness, as well as indirect improvements through expanded ART access. We found that TB programme improvements as well as ART expansion can make important and complementary contributions to TB control. Improvements made to the TB programme act to lower TB prevalence and mortality, with secondary reductions to TB incidence. ART expansion, by lowering the vulnerability of the HIV-affected population to the risk of TB disease after infection [9] and preventing further HIV transmission [8], facilitates control of TB over a longer time horizon.

Of the different dimensions of the direct TB programme changes, we found that expanding programme coverage would have the largest impact on TB control. Importantly, we found that improvements to both the TB and HIV programmes are likely to produce complementary benefits for TB control since these interventions target different drivers of the TB epidemic. Model projections demonstrate the potential for improvements in tuberculosis programmes for reducing the burden of MDR-TB; we note that these projections assume that the biological costs associated with resistance are fixed at a single value. If higher fitness resistant strains are preferentially transmitted, then these simulations will underestimate the effort necessary to reduce the burden of MDR-TB [25].

This work expands upon previous modelling studies in two significant ways. First, the introduction of new diagnostics such as Xpert and the prospect of novel treatment regimens highlight the rapid change in the tools available for strengthening different dimensions of TB programmes. By considering alternative target elements for improving TB control programmes, we were able to quantify the relative magnitude of improvements that could be achieved by each approach. Second, understanding of the impact of ART on reducing the risk of TB as well as on reducing HIV transmission has improved [8] since earlier modelled analyses were published. We incorporated this new evidence into our estimates, capturing lowered risks of TB among people on ART and reduced rates of HIV transmission to their sexual partners. As demonstrated in the sensitivity analysis, the effect of ART in reducing HIV transmission is large, and particularly important over the long term, with the effect of ART scale-up on TB incidence and mortality rates growing progressively over time. In contrast, despite recent evidence on diagnostics with improved performance characteristics, improving access to services appears to represent the area with greatest potential for improvement.

Currie *et al.* [5] modelled the TB–HIV epidemics in three African countries and found that TB programme expansion was the most effective means of controlling the TB epidemic over 10 years, whereas ART coverage needed to substantially increase relative to baseline levels before significant reductions in TB incidence could occur [8]. At the time, trial evidence on the reduction in HIV transmission following ART initiation was not available. By incorporating this new evidence, our work suggests that ART expansion is an effective way of reducing HIV incidence, and thus HIV-associated TB incidence, over the long term. Williams *et al.* [3] considered the impact of different ART initiation times among HIV-positive people on control of the TB epidemic in nine African countries. Their work does not investigate the potential effects of TB programme improvements. Our results were consistent with their work in concluding that frequent HIV testing combined with immediate treatment initiation will substantially decrease the burden of HIV-associated TB over the long term. Our work also showed that delaying TB programme improvement erodes these potential benefits, complementing the results of the prior analyses that examined how delaying ART expansion will reduce its potential impact.

Recently, Dodd *et al.* examined the consequences of expanding ART guidelines [26], and found that increased access to ART may paradoxically lead to a rebound of TB incidence in the long term owing to an increased life expectancy in people living with HIV. Their work assumes that the TB protection afforded by ART declines over time, and that HIV incidence is independent of ART coverage. When we also assumed an HIV incidence independent of ART coverage, we found that TB incidence declined more slowly than in our main results, but did not rebound (electronic supplementary material, figure S3), suggesting that a declining immunological response to ART underlies this rebound in TB. This difference in predictions highlights the importance of the durable effects of ART to long-term TB outcomes.

For the scenarios describing programme expansion in one or more dimension, we attempted to define the maximal level of expansion to represent the limit of what might be possible. The choice of these limits is essentially arbitrary, but the range defined by these limits and continuation of the status quo should encompass the spectrum of options relevant for decision-makers, and so readers can interpret the results in light of their own beliefs about what is plausible. Our analysis neither estimated resource consumption nor calculated summary measures of health benefit (e.g. DALYs averted), and was not intended to describe the optimal intervention approach. Instead, our aim was to consider the effects of various TB control approaches, given our improved understanding of intervention effects and the current salience of ART as a TB control intervention. We also did not attempt to model additional coordination between the HIV and TB programmes, which had been posited to be an important structural intervention for controlling the TB epidemic in South Africa [27,28]. Recently, the South African national HIV programme announced a change in the eligibility threshold for initiating ART, broadening eligibility from a CD4 count of 350 cells per µl to a count of 500 cells per µl. We expect that this policy change will realize some of the TB-related benefits of expanding ART demonstrated in our results, though less than would be achieved by the policy we investigated, which assumed universal ART eligibility and scaled-up testing programmes.

Our findings suggest that, despite the considerable enthusiasm generated by improved TB diagnostics and novel TB treatment regimens, it is programme coverage expansion that would produce the largest impact on various metrics of TB burden, whereas diagnosis and treatment improvements each would produce no more than half of the improvement possible with increased coverage. Although it might be easier to focus attention on those patients who already have access to the health system, increasing access to extend coverage may be the most effective means of controlling the TB epidemic. This finding of course needs to be balanced against the fact that increasing coverage becomes costlier and more logistically challenging as higher levels of coverage are attained, especially in a resource-limited setting like South Africa. Achieving high levels of coverage may require increased intensity of existing intervention strategies (with higher marginal cost), but may also require novel intervention strategies (or technologies) with substantially increased unit costs compared with current approaches. As a consequence, the results of this analysis, describing magnitude of impact on various epidemiological outcomes, should not be taken as a proxy for the relative priority of these different interventions within a resource allocation framework.

Our results further suggest that ART expansion can be a more effective route to controlling HIV-fuelled TB epidemics when it is combined with TB programme improvements. These two interventions work in complementary ways, with ART expansion protecting HIV patients from developing TB as well as dramatically lowering transmission of HIV in the population, and TB programme improvements reducing the prevalence and mortality among TB patients. The joint impact of these interventions substantially exceeds that of each individual intervention on various metrics of TB burden.

Our findings also underscore the importance of considering the long-term impacts of different interventions, rather than just the short-term ones; indeed, while we found that TB expansion would outperform ART expansion in the first 5 years after its introduction, the benefits of ART would accrue over time, suggesting that control of the HIV epidemic represents a critical dimension in an overall strategy to improve long-term TB outcomes in settings experiencing a high burden of both diseases.

## 4. Material and methods

### 4.1. Model structure

The model divides the population into a set of discrete compartments and simulates transitions between these compartments representing infection, progression and treatment for TB and HIV. In addition to the core model structure capturing TB infection and natural history, TB compartments are further subdivided to track the development and propagation of TB drug resistance phenotypes, and to track prior TB treatment history. A detailed description of model states and parameters is provided in the electronic supplementary material.

### 4.2. Model calibration

**Figure 5.**
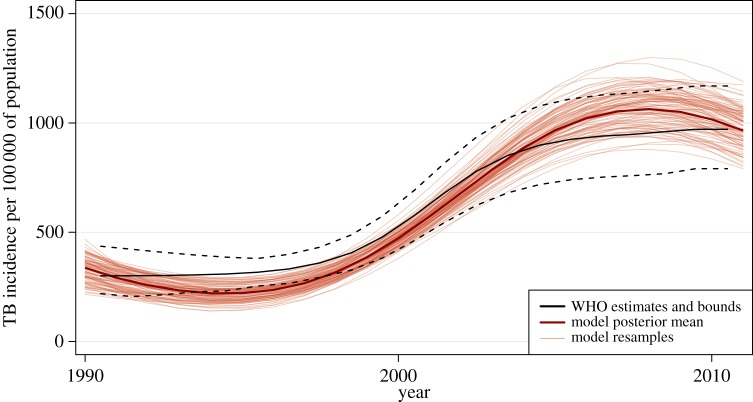
An illustration of the results of model calibration based on fit to available data. (Online version in colour.)

We sampled the parameter value space defined by prior distributions specified around all model parameters and calibrated the model as described in [19]. Because the model runs deterministically once the parameters are chosen, we defined a likelihood function to estimate the goodness-of-fit of a particular parameter set to the calibration data, which included notification data, historical TB incidence and prevalence (including MDR-TB) and HIV prevalence. In the likelihood function, we considered the prevalence and incidence of TB at 5-year intervals between 1990 and 2010, the prevalence of HIV at 3-year intervals between 2002 and 2008, the prevalence of MDR-TB in 2002, and the notification data every year from 1990 to 2010, as independent outcomes. We constructed a probability distribution around each one (a beta distribution around the prevalence of MDR-TB and of HIV, and a normal distribution around the other outcomes) and multiplied the resulting density values to obtain the final likelihood. Figure 5 illustrates the result of model calibration for one outcome, TB incidence.

We used the sampling importance resampling (SIR) approach [22]. In the first (‘sampling’) stage, we created 500 000 parameter sets sampled from the prior distribution using Latin Hypercube Sampling [29]. For each of these sets, we computed the likelihood by comparing the model outcomes to calibration data. In the second (‘resampling’) stage, we created a new sample to represent the posterior distribution of model parameters by assigning weights to each parameter set proportional to its likelihood. Electronic supplementary material, table S3 presents the range, posterior mean and 95% credible intervals of the posterior distribution of each model parameter.

### 4.3. Modelled scenarios

Our baseline scenario assumed the maintenance of current TB and HIV control policies and programmes. In this scenario, ART coverage among eligible groups, modelled as all individuals with CD4 < 350 cells per µl or active TB, is projected to increase smoothly using a logistic growth curve until it reaches a plateau at 80% in 2017, and to stay essentially constant thereafter.

The first set of alternative scenarios (‘TB programme improvement’) represents accelerated progress in three dimensions of traditional TB control programmes:

(1) Improved healthcare coverage for individuals experiencing TB symptoms, operationalized as reducing the average delay from disease development to clinic attendance to a minimum of one-third of its current value.
(2) More accurate TB diagnosis, operationalized by replacing a proportion of tests using smear microscopy with a more sensitive and specific diagnostic test, the GeneXpert MTB/RIF assay. At the maximum, this scenario would have 100% of TB diagnosis undertaken with an Xpert-based algorithm.
(3) Improved TB treatment, operationalized as
  (i) reduced primary default after diagnosis (to an extremum of 0% primary default),
  (ii) improved cure rates (to an extremum of 100% cure),
  (iii) improved identification of treatment failure (to an extremum of 100% identification of treatment failure), and
  (iv) reduced treatment default (to an extremum of 0% treatment default).

As a consequence of the multiple changes described for improved TB treatment, the maximal level for this dimension would imply that all individuals receiving a positive TB diagnosis would be successfully treated for TB.

We parametrized each TB programme expansion scenario by three numbers, each between 0 and 1, where a 0 corresponds to the baseline value for each of the three dimensions described above (coverage, diagnosis and treatment) and a 1 corresponds to the best possible value for this dimension.

The second set of alternative scenarios (‘ART expansion’) represents the combination of universal ART eligibility for HIV-positive patients with an increased ART initiation rate for all HIV-positive individuals. We operationalize this increase by adding 0.5 per year to the rate of ART initiation achieved in the baseline scenario, separately for each CD4 category (high, medium and low) as well as the category of those on TB treatment.

We assumed that any changes in current programme strategy would be introduced progressively over the period between 2012 and 2014 and then maintained until 2032. The speed of intervention improvements was varied in our sensitivity analysis.

### 4.4. Outcomes

To compare the overall potential for direct and indirect interventions to control TB, we examined the levels of improvement that would be required along each of the three dimensions of improved TB control to attain a comparable benefit to that attained through ART programme expansion.

To find the set of points (*X*_1_, *X*_2_, *X*_3_) that matched the ART intervention for a particular outcome, we fixed a resolution (usually 1/128) and then created a two-dimensional grid in which two of the dimensions were equally spaced from 0 to 1 at this resolution. We then filled this grid with values of the third coordinate that yielded a matching value of the outcome by performing root-finding along that coordinate with the secant method, relying on the fact that the outcome varies monotonically with each dimension to bound the values from above and below. This procedure produces the isocline plots shown in figure 3.

### 4.5. Sensitivity analysis

We computed partial rank correlations using the *prcc* function in the *epiR* package for the *R* statistical computing language [30]. The partial rank correlation is an estimate of the relative influence of each model parameter on the outcome of interest when all the other parameters are being held constant, and is always between −1 and 1.

We estimated the effects of the rate of TB programme scale-up by changing the transition from initial levels to target levels of the set of parameters describing TB programme improvements to take place over 5 or 10 years instead of 2 years. For each parameter, the transition was modelled by a linear function. We estimated the effects of the parameters governing ART effectiveness and ART coverage expansion by halving their values.

To compare our results with the approach adopted by Dodd *et al.* [26], in which one of the key projections assumes that HIV incidence does not change over time, we modified our model by adopting the HIV incidence trend from the baseline scenario and using this for each of the interventions. Accordingly, this reduces the impact of ART on HIV control, which results in a higher incidence and mortality of TB. The approach our model normally uses is to take the time-varying contact rate from the baseline scenario and use that to infer the HIV incidence by taking the average of the distribution of HIV-positives across disease and treatment stages weighted by their relative infectivity. This results in a significantly lower TB incidence relative to the baseline scenario when ART is scaled up aggressively.

## Appendix A. Model overview and structure

Analyses were conducted using a dynamic compartmental model of TB and HIV in adult populations. The model simulates transitions between health states deterministically, recalculating the population distribution across states in discrete monthly time steps. The model was constructed and run using *R* statistical computing software [30]. The model follows the conventions of earlier TB models [31–37], with additional detail to accommodate evaluation of alternative TB programme improvement strategies and ART expansion. The model structure is defined by a set of core TB states, and these states are further subdivided to account for: (i) aspects of HIV infection, progression and treatment relevant to TB epidemiology, (ii) multiple circulating TB strains, with different drug resistance profiles, and (iii) tracking of TB treatment history. Electronic supplementary material, figure S1 illustrates the model structure, described in further detail below.

**A.1. Core tuberculosis states**

The core TB states capture important features of TB transmission, natural history and treatment. Eight states are included. Individuals who have never been infected reside in the susceptible state. Those who are infected but do not have active disease are in the latent infection/recovered state. Active disease is categorized as smear-negative or smear-positive. Smear-negative or smear-positive active cases may be treated either through the national TB control programme (DOTS), or through providers outside of the national programme (non-DOTS).

**A.2. Human immunodeficiency virus subdivisions**

HIV co-infection can alter the rate of progression of TB disease, with HIV-infected individuals having a higher probability of primary progressive TB upon initial infection [38,39], a higher rate of breakdown from latent infection to active TB [40], a lower probability of smear-positivity among those with active disease [41–43] and higher mortality rates [41,44,45]. The HIV part of the model draws on structure and assumptions from an array of published HIV models [46–49]. There are seven HIV subdivisions. Individuals may be HIV-negative, they may be in one of three categories reflecting untreated HIV infection with a specified CD4 cell count (greater than 350, 200–350 and less than 200 cells per µl), or they may be receiving ART in one of three categories distinguished by the CD4 count at treatment initiation.

**A.3. Drug resistance subdivisions**

Five subdivisions were created to account for differences in drug resistance among circulating TB strains, including (i) pan-sensitive TB, (ii) isoniazid (INH) mono-resistant TB, (iii) rifampicin (RIF) mono-resistant TB, (iv) resistance to both isoniazid and rifampicin (MDR-TB), and (v) resistance to isoniazid and rifampicin plus one or more second-line drugs (MDR+/XDR-TB).

**A.4. Treatment history subdivisions**

A final subdivision of model states distinguishes treatment-naive from treatment-experienced individuals, as diagnostic algorithms may dictate different confirmatory tests depending on an individual's history of prior treatment.

**A.5. Summary of model structure**

At any point in time, all individuals in the model are categorized by the combination of their TB status and their status with respect to each of the three subdivisions. Thus, each of the eight core states is ‘exploded’ into 70 unique substates (resulting from seven HIV categories × five drug resistance categories × two treatment history categories), which yields a total of 8 × 70 = 560 unique compartments in the model. Fifty-six of these 560 compartments, namely those corresponding to susceptible individuals with a TB strain having a specific drug resistance profile, are null.

## Appendix B. Transitions between model states and subdivisions

The model transitions may be represented by a set of difference equations. Electronic supplementary material, table S1 defines the general notation used in the formal description of the model that follows.

**B.1. Transitions between core tuberculosis states**

We begin with a set of model equations that describe changes in the population distribution across the eight core TB states between one timestep and the next. In the following equations, *X_i_* indicates the number of residents in state *i* at time *t*, and  (with a dot above the *X*) indicates the number of residents in state *i* at time *t* + 1.

The total population is given by

Individuals enter the model in the susceptible state (*X*_1_), where they face a time-varying risk of TB infection. Formally, the force of infection, *λ_t_*, describes the hazard rate (at time *t*) by which a susceptible individual acquires TB. The population is assumed to mix randomly with density-independent contact rates, so transmission is modelled as frequency-dependent. The force of infection allows for varying infectivity across different categories of disease, and for temporal trends in contact rates, which yields the following formulation in the simple case of a single circulating TB strain:

where *β*_t_ is the transmission parameter for those with untreated, smear-positive, active disease at time *t*, and *q_i_* is the infectivity of individuals in core state *i* relative to those with untreated, smear-positive active disease.

Upon infection, individuals progress either to latent infection (*X*_2_) or directly to active disease. Individuals with latent infection may subsequently progress to active TB, or they may be re-infected at a rate that is subject to the partial immunity conferred by an existing infection. Active disease is categorized as smear-negative (*X*_3_) or smear-positive (*X*_4_). Smear-negative cases may progress to smear-positive, and all individuals with active disease may spontaneously self-cure, which returns them to the latent/recovered state. An individual with active disease can be diagnosed as a TB case, according to the characteristics of the diagnostic algorithm, and initiated on treatment. Treatment may be provided either through DOTS, the national TB control programme (*X*_5_ for smear-negative and *X*_7_ for smear-positive cases), or through non-DOTS providers outside of the national programme (*X*_6_ for smear-negative and *X*_8_ for smear-positive cases). Treated individuals may complete treatment, default (returning to active disease) or die. Those who complete treatment are categorized as failures (returning to active disease) or cures (returning to the latent/recovered state). In addition to these transitions, all individuals in the model are subjected to a background mortality rate which is updated in each time step based on demographic data, and to TB-related mortality specific to each active disease state.

**B.2. Transitions between human immunodeficiency virus subdivisions**

Rates of transition from one HIV subdivision to another are based on estimates of HIV incidence, disease progression and treatment initiation (see appendix C.4 and electronic supplementary material, table S3). These rates are assumed independent of core TB states and other subdivisions. HIV incidence is modelled as a transition from the HIV-negative category to the HIV-positive, CD4 > 350 category, with time-varying incidence rates defined as exogenous model parameters. HIV-positive individuals not on ART may progress over time to lower CD4 counts. Untreated HIV-positive individuals transition onto ART at rates specific to CD4 category, which are allowed to vary over time to capture changing eligibility criteria and coverage of testing and referral. HIV-related mortality occurs at rates specific to each subdivision. Certain parameters governing the natural history of TB vary with respect to HIV status, as indicated in electronic supplementary material, table S3.

**B.3. Transitions between drug resistance subdivisions**

Transitions between TB strain subdivisions occur through infection, superinfection and acquired resistance. First, we elaborate the specification for the force of infection to allow for multiple circulating strains distinguished by their drug resistance profiles. Individuals may be infected by any of the five types of strains. When calculating the force of infection for a particular strain (*λ*_s_ for strain *s*), we allow for differential fitness across strains, for example indicating lower transmissibility among drug-resistant strains relative to drug sensitive ones. The total force of infection is a weighted sum of the five strain-specific forces of infection. The general formulation for the force of infection is thus given by

where *r*_s_ is the relative reduction in fitness for strain *s* compared with the corresponding pan-sensitive strain. An individual in the susceptible state who is newly infected with TB transitions to the subdivision of the infecting strain. An individual with latent TB who is superinfected by a different strain transitions to the subdivision of the superinfecting strain. Following Lipsitch *et al.* [50], we allow for superinfection by the same strain in order to preserve model neutrality with respect to strain distribution.

Individuals may also develop acquired drug resistance during TB treatment. Individuals with pan-sensitive TB can develop mono-INH resistance, mono-RIF resistance or MDR-TB directly. Individuals with mono-INH or mono-RIF can develop MDR-TB, and individuals with MDR-TB can develop MDR+/XDR-TB. Cases of acquired resistance arise as individuals default from or fail treatment, with rates of acquiring resistance specified for each combination of current strain and specific treatment regimen (electronic supplementary material, table S3).

**B.4. Transitions between treatment history subdivisions**

Individuals enter the model in the treatment-naive category. Treatment-naive individuals move into the treatment-experienced category upon the first transition out of any of the TB treatment states (*X*_5_, *X*_6_, *X*_7_ or *X*_8_) in the core model.

## Appendix C. Model parametrization

**C.1. Initialization**

The model was used to estimate TB prevalence and incidence starting in 1950 onwards, with this long historical projection allowing the simulation of a realistic TB epidemic as well as providing prevalence and incidence estimates for the recent past to compare with independent data in the calibration procedure. First, we simulated a virgin epidemic, in which one infectious source case is introduced into a population of susceptibles. This epidemic was run to equilibrium, which was assumed to represent the starting conditions in 1950. The model was then run from 1950 through the end of 2011 to produce a historical time trend in TB epidemiology, with time-varying parameter values capturing changes in birth rates, background mortality rates, TB contact rates, access to TB and HIV treatment interventions, and treatment success and default rates. Electronic supplementary material, table S3 summarizes estimates and ranges for all model parameters. Following is a description of key data sources used to derive these values and ranges.

**C.2. Demographics**

Historical estimates and future projections for population growth were obtained from the United Nations Population Division [51]. Historical estimates for mortality excluding HIV were obtained from the World Health Organization (WHO), and future background mortality was held constant at current values.

**C.3. Tuberculosis epidemiology, diagnosis and treatment**

Estimates for transition rates between TB-related health states were drawn from the literature and chosen to be consistent with prior TB modelling work [25,33–35,52,53]. ART delays the immunosuppression associated with HIV, thereby reducing the effect of HIV on TB disease progression. We operationalized this as an ART effectiveness parameter (*z*); the values of TB natural history parameters for individuals on ART were calculated as weighted sums of parameter values for HIV-negative and untreated HIV-positive individuals, with weights *z* and 1 − *z,* respectively.

Individuals receiving TB treatment were assumed to have reduced infectiousness compared with untreated individuals, with the reduction in infectiousness approximated as the complement of the failure probability for each regimen–strain pair. Diagnostic algorithms were based on current practice and on WHO guidelines for Xpert implementation [54]. Values for the sensitivity and specificity for each diagnostic test were derived from the published literature [55–57]. As the model distinguishes between smear-negative and smear-positive TB, the sensitivity of smear was defined as 0% and 100%, respectively, for these two groups. As sputum culture is considered the gold standard for diagnosis the sensitivity of this test was assumed to be 100%. Few data are available on the percentage of individuals testing negative on smear microscopy who subsequently have this diagnosis confirmed by sputum culture. Dowdy *et al.* [52] estimated this percentage as 5% and 37% for treatment-naive and treatment-experienced individuals, respectively, based on 2004 South African data. It is likely that access to sputum coverage will have risen since then, and we assumed starting values for these parameters of 20% and 80%, respectively. In addition, 80% of individuals who are diagnosed positive with a history of prior treatment were assumed to receive drug susceptibility testing (DST).

Parameters relating to treatment programme coverage and performance were based on routine monitoring data aggregated by the WHO Stop TB Department [58]. Access to DOTS TB programmes (parametrized as the rate at which those with active TB attend a health centre providing TB diagnosis and treatment) was estimated from reported trends in the case detection rate (CDR). First, a simple time trend was fit to national CDR data using a logistic regression model. As the CDR more closely approximates a probability rather than a rate, we transformed the predicted CDR (CDRp) to calculate the attendance rate, whereby rate = 1 − exp(−CDRp). For the pre-1990 period, the rate of attendance for DOTS diagnosis was assumed to increase from zero to the 1990 value over a 4-year period. For future years, the attendance rate was held constant at the most recent value for which data were available. The imperfections of the CDR as a measure of the probability of detection are well understood [59], and this uncertainty was reflected in the analysis by assuming a wide prior distribution for the attendance rate, with a range spanning from zero to two times the point estimate. There is little information on non-DOTS diagnosis, but this was assumed to start earlier (1970) and to continue at a low level in the future (rate of 0.2 per year, also varying within a range spanning zero to two times the point estimate). The volume of non-DOTS care was calibrated to produce observed drug resistance levels.

Rates of treatment default were based on reported programme outcomes [58] and calculated in a similar fashion to the attendance rate, by fitting a simple time trend to the national programme data using a logistic regression model, and transforming the estimated probability of default to obtain the annualized default rate. TB-specific excess mortality rates were assumed to persist for the first two months of treatment before dropping to zero, and the treatment mortality rates produced by this assumption were consistent with reported programme outcomes.

The probability of treatment success (probability of cure or completion among all individuals finishing a treatment regimen) are determined by the appropriateness of the drug regimen as well as other characteristics of the treatment programme—such as quality of adherence support—which might change over time. To capture the influence of these other programme characteristics, we assumed that the efficacy of the first-line regimen in pan-sensitive TB was equivalent to the fraction of all individuals cured or completing treatment estimated from national programme data. This was operationalized as a time trend fit to the observed data in a logistic regression model. The probabilities of treatment success for other strain–regimen combinations were assumed to be fixed proportions of this value, shown in electronic supplementary material, table S3.

We assumed that diagnosis and treatment were more limited in the early years of TB control programmes. This assumption was operationalized in the model as a linear increase in the availability of culture, DST and second-line regimens over the past 20 years, from an initial scenario in which there was no access to advanced tests or second-line regimens.

Little information is available to estimate rates of acquired resistance by regimen and initial strain. We based our estimates on data reported in Lew *et al.* [60], adjusted for the prevalence of resistance to other first-line drugs (streptomycin, ethambutol) not tracked in the model (values shown in electronic supplementary material, table S3).

**C.4. Human immunodeficiency virus epidemiology and treatment**

Estimates for HIV incidence and ART coverage were obtained from UNAIDS (2015, unpublished data). For future years, HIV incidence was assumed to decline at an exponential rate estimated from the past 7 years of incidence data. Untreated HIV-positive individuals in the model transition onto ART at rates calculated to match national reporting data on ART programme scale-up. ART coverage (the fraction of eligible individuals receiving ART) was assumed to increase from current levels to the WHO universal access target of 80% coverage [61] over the course of 10 years. Early HIV treatment guidelines suggested a CD4 count criterion of less than 200 cells per µl for initiating ART [62], whereas the 2010 revisions to the guidelines have raised this CD4 criterion to less than 350 cells per µl [63]. For this reason, all ART initiations prior to 2010 were assumed to come from the CD4 < 200 group, and for 2010 onwards, the fraction of HIV initiations coming from the 350 > CD4 > 200 group was assumed to rise such that by 2015 individuals in the 350 > CD4 > 200 and CD4 < 200 groups would have equal probability of initiation on ART. The most recent guidelines, adopted after this work was underway, suggest that ART be initiated in all HIV-positive individuals with a CD4 count less than 500 cells per µl [64]; we do not model individuals with CD4 count above and below this new threshold separately. Estimates for HIV-specific mortality rates (with and without ART) were drawn from the literature [65–70].

## Appendix D. Model calibration

We adopted a Bayesian approach to calibrate the model, following the prior work of Raftery and co-workers [20,21]. The approach enables the synthesis of multiple sources of information on the values of model outputs, and allows for characterization of the uncertainty in model results using Bayesian posterior intervals and similar metrics.

The disease model (*M*) can be considered a deterministic mapping from the parameter space of the model inputs (*Θ*) to that of the model outputs (*Φ*), such that *M* : *θ* → *φ*. For some of these outputs (*φ*_1_), we have external data (*X*) related to *φ*_1_ through a defined probability model. An example of *φ*_1_ would be model projections of MDR-TB prevalence for 2010, and an example for *X* would be the estimate for MDR-TB prevalence obtained from a population-based survey conducted in the same year. For other outputs (*φ*_2_)—generally those we would like to make inferences about—we have no external data, but can estimate their distribution based on the prior information about *θ* and *φ*_2_, relying on the deterministic disease model to link these three sets of parameters. As we have probabilistic prior information on *θ* and *φ*_1_, we can use this information to estimate the posterior density of *θ*

where Pr(*θ*|*X*) is the prior distribution of the model inputs, and *L*(*θ*|*X*) is the likelihood function for *θ* constructed with the external data *X*. While this likelihood function cannot be estimated directly, we can transform *θ* into the output parameter space to estimate the likelihood

Having obtained a posterior distribution for the model inputs, we can then estimate the posterior density of *φ*_2_ through the model, as *M*(Pr(*θ*|*X*)). An analytic solution can be difficult or impossible to calculate for disease models of moderate or greater complexity, but the posterior distributions can be approximated using numerical methods. Following Alkema *et al.* [20], we used an SIR algorithm [22].

— We quantified the prior uncertainty for each model parameter. Each range was assumed to represent the 95% CI for a lognormal distribution (for parameters defined over positive numbers, e.g. rates) or logit-normal distribution (for parameters defined over the interval [0,1], e.g. probabilities).
— We constructed a likelihood function to calibrate the model, based on (i) WHO estimates [58] for TB prevalence and incidence in 1990, 2000 and 2010, (ii) results from a 2002 TB drug resistance survey [71], and (iii) HIV prevalence estimates from national seroprevalence surveys in 2002, 2005 and 2008 [72]. These data are summarized in electronic supplementary material, table S5. A normal likelihood was adopted for each TB prevalence and incidence estimate, centred at the reported value and with variance calculated from the width of the reported confidence intervals. A multinomial likelihood was adopted for the distribution of TB drug resistance (summarized into four categories: pan-sensitive TB, mono-INH resistant, mono-RIF resistant and MDR-TB), with separate likelihoods constructed for treatment-naive and treatment-experienced cases, and assuming a design effect of 2 for the survey sample. A binomial likelihood was adopted for the HIV prevalence estimates, constructed using the survey results. These likelihood functions were assumed to be mutually independent, and multiplied to create a joint likelihood function.
— We used Latin Hypercube Sampling to draw 500 000 random parameter sets, and a separate simulation conducted for each of these parameter sets. A likelihood statistic was calculated for each of these model runs by applying the joint likelihood function to the model outputs produced by a particular parameter set.
— We then resampled the 500 000 parameter sets from the first-stage sample with replacement to create a final array of parameter sets, using the likelihoods as sampling weights. A sample size of 10 000 was used for this second sample.
— We calculated results by running the model for the resampled array of parameter sets. For each quantity of interest from the model, the point estimate was calculated as the mean of the results for the second stage sample, and 95% posterior intervals (the Bayesian equivalent of confidence intervals) calculated from the 2.5th and 97.5th percentiles of the simulation results for each quantity of interest.

Figure 5 shows the results of the calibration for TB incidence, overlaid with the WHO estimates.

## Appendix E. Sensitivity analyses

We adopted three approaches to investigate the sensitivity of our results to changes in model inputs.

**E.1. Ranking variability analysis**

In order to understand the robustness of our qualitative conclusions, we examine the resampled parameter sets individually to determine whether they rank pairs of interventions consistently or inconsistently with the posterior mean for each outcome. The fraction of the probability mass assigned to the resampled parameter sets which produce a consistent ranking provides a measure of the robustness of our conclusions.

**E.2. Analysis of partial rank correlation coefficients**

Partial rank correlation coefficients (PRCCs) represent a complementary approach for investigating uncertainty, providing information on the relative influence that individual parameters have on model outcomes based on the results of a probabilistic sensitivity analysis [35,73,74]. We calculated PRCCs using the resampled parameter sets produced by the calibration procedure. Results for the 10 parameters having the greatest influence on the TB incidence reduction over 20 years are shown in electronic supplementary material, figure S2.

**E.3. Fixed incidence instead of fixed contact rate**

Our model's baseline HIV incidence is normally used to compute a time-dependent contact rate, which is then used in all alternative scenarios to compute the new incidence. Both of these are computed using the equation

where *I_t_* is the incidence at time *t*, *C_t_* is the contact rate, *r_j_* is the infectiousness of the *j*th HIV state relative to the untreated HIV-positive state with CD4 > 350, and *X_jt_* is the fraction of people in HIV state *j* at time *t*. In order to compare our model with that of Dodd *et al.* [26], we use the baseline incidence in all of our scenarios, effectively turning off the impact of ART expansion on HIV incidence.
